# Overall survival after relapse in high-risk neuroblastoma: an adjusted indirect comparison of post-relapse dinutuximab beta, historical controls, and relapse after first-line dinutuximab beta maintenance

**DOI:** 10.3389/fonc.2026.1933438

**Published:** 2026-10-07

**Authors:** Holger N. Lode, Przemysław Holko, Aleksandra Wieczorek, Katarzyna Śladowska, Nikolai Siebert, Dominique Valteau-Couanet, Alberto Garaventa, Adela Cañete, John Anderson, Isaac Yaniv, Shifra Ash, Lucas Moreno, Juliet Gray, Roberto Luksch, Genevieve Laureys, Cormac Owens, Carla Manzitti, Sascha Troschke-Meurer, Paweł Kawalec, Ruth L. Ladenstein

**Affiliations:** 1 Department of Pediatric Hematology and Oncology, University Medicine Greifswald, Greifswald, Germany; 2 Department of Nutrition and Drug Research, Institute of Public Health, Faculty of Health Sciences, Jagiellonian University Medical College, Krakow, Poland; 3 Paediatric Haematology Oncology, Jagiellonian University Medical College, Krakow, Poland; 4 Children and Adolescent Oncology Department, Gustave Roussy, Paris-Sud University, Paris, France; 5 Oncology Unit, Scientific Institute for Research, Hospitalization and Healthcare (IRCCS) Istituto Giannina Gaslini, Genoa, Italy; 6 Hospital Universitario y Politecnico La Fe, Valencia, Spain; 7 University College London (UCL) Great Ormond Street Institute of Child Health, London, United Kingdom; 8 Schneider Children’s Medical Center of Israel, Sackler Faculty of Medicine Tel Aviv University, Petach Tikva, Israel; 9 Schneider Children's Medical Center of Israel, Petach Tikva, Israel; 10 Division of Pediatric Hematology & Oncology, Vall d’Hebron Barcelona Hospital, Barcelona, Spain; 11 Centre for Cancer Immunology, University of Southampton, Southampton, United Kingdom; 12 Pediatric Oncology Unit, Fondazione IRCCS Istituto Nazionale dei Tumori, Milan, Italy; 13 Department of Pediatric Hematology/Oncology and Stem Cell Transplantation University Hospital Ghent, Ghent, Belgium; 14 Department of Haematology and Oncology, Children's Health Ireland, Dublin, Ireland; 15 Centrum HTA Sp. z o.o., Kraków, Poland; 16 Department of Paediatrics, Saint Anna Children’s Hospital, Medical University, Vienna, Austria; 17 Department of Pediatrics, Medical University Vienna, Vienna, Italy

**Keywords:** adjusted indirect comparison, anti-GD2 immunotherapy, dinutuximab beta, high-risk neuroblastoma, overall survival, relapsed neuroblastoma

## Abstract

**Objective:**

To compare overall survival (OS) in patients with relapsed neuroblastoma treated with dinutuximab beta (DB) after relapse with that of two comparator populations: historical controls with no prior anti-disialoganglioside-2 exposure and patients who relapsed after first-line DB maintenance immunotherapy. The objectives were to evaluate the effectiveness of DB in the relapsed setting and to assess a potential legacy effect of prior first-line therapy.

**Methods:**

This unanchored indirect comparison used individual patient data from three cohorts: historical controls with no prior anti-GD2 therapy (N/N), patients who relapsed after first-line DB maintenance immunotherapy and did not receive subsequent DB (DB/N), and patients treated with DB after relapse without prior exposure (N/DB). Harmonized eligibility criteria were applied across cohorts, and follow-up was aligned using a common post-relapse index date. Adjusted comparisons were performed using propensity score reweighting. Overall survival was measured from the observed (N/DB) or imputed (N/N and DB/N) post-relapse index date and analyzed using weighted Kaplan–Meier methods and weighted Cox proportional hazards models, with hazard ratios (HR) reported. For the N/N and DB/N cohorts, the post-relapse index date was imputed using a quantile regression model estimating the interval from first relapse to initiation of DB, fitted to data from the N/DB cohort.

**Results:**

A total of 329 patients were assessed, of whom 266 were included in the weighted analyses. Baseline imbalances in age at relapse, time from diagnosis, MYCN status, and International Neuroblastoma Staging System (INSS) stage were largely resolved after weighting. No evidence of a legacy effect of first-line DB exposure was observed, as OS did not differ significantly between patients who relapsed after first-line DB maintenance immunotherapy and historical controls (HR = 1.26, 95% CI 0.70–2.26; p=0.437). In contrast, DB administered after relapse was associated with significantly improved OS compared with both historical controls (HR = 0.53, 95% CI 0.35–0.81; p=0.003) and patients who relapsed after first-line DB maintenance immunotherapy (HR = 0.42, 95% CI 0.25–0.72; p=0.001). These findings were consistent across sensitivity analyses.

**Conclusion and limitations:**

This adjusted indirect comparison demonstrated that DB administered after relapse was associated with significantly improved OS compared with both historical controls and patients who relapsed after prior first-line DB maintenance immunotherapy. No evidence of a legacy effect of first-line DB immunotherapy was identified. These findings support the clinical relevance of DB in the relapsed setting and indicate that prior first-line DB maintenance alone was not associated with a persistent post-relapse survival advantage. The study did not evaluate DB retreatment after prior first-line exposure.

## 1 Introduction

Neuroblastoma is the most common extracranial solid malignancy in children and remains a leading cause of cancer-related mortality in the pediatric population. Despite substantial therapeutic advances, outcomes for patients with high-risk disease remain poor, particularly after relapse, which continues to be the primary cause of treatment failure in this population (1, 2).

The introduction of anti-disialoganglioside 2 (GD2) monoclonal antibodies has represented a major advance in the treatment of high-risk neuroblastoma. In the post-consolidation setting, GD2-directed immunotherapy has improved event-free survival (EFS) and overall survival (OS) and is now an established component of first-line treatment. This benefit has been demonstrated in both Children’s Oncology Group studies of post-consolidation GD2-directed immunotherapy and the SIOPEN phase 3 trial evaluating dinutuximab beta (DB)–based maintenance therapy (3–5).

Relapse remains common in high-risk neuroblastoma, and treatment strategies for relapsed or refractory disease remain heterogeneous, with no universally accepted standard for relapse management. However, chemoimmunotherapy, including anti-GD2–based combinations, is increasingly supported as part of reinduction therapy (2). DB-based chemoimmunotherapy has shown encouraging activity in heavily pretreated patients, while evidence syntheses and real-world multicenter data support the clinical relevance of anti-GD2–based combinations and suggest broadly consistent activity across chemotherapy backbones (6–8). Randomized phase 2 evidence from the ITCC-SIOPEN BEACON Immuno trial further supports the addition of DB to temozolomide-based chemotherapy, with improved objective response and progression-free survival, although interpretation of OS was limited by crossover (9).

Other GD2-directed salvage approaches, such as naxitamab plus granulocyte-macrophage colony-stimulating factor (GM-CSF), have also demonstrated clinically meaningful activity, further supporting GD2 as a relevant therapeutic target beyond first-line therapy (3, 10). The increasing use of anti-GD2 immunotherapy in the frontline setting raises the unresolved question of whether prior DB exposure influences relapse biology and post-relapse prognosis. This question is clinically relevant given concerns regarding late relapse, central nervous system relapse, and mechanisms of immune evasion or treatment resistance (3, 11). Evidence on anti-GD2 retreatment remains limited and largely retrospective or non-randomized (12). Although available data suggest that anti-GD2–based therapy may retain activity in some previously exposed patients, the magnitude of benefit after frontline exposure remains uncertain, and further evidence is needed specifically in patients previously treated with anti-GD2 therapy (6, 12).

Direct comparative evidence on DB retreatment in patients previously treated with anti-GD2 antibodies is lacking. In this context, indirect comparison methods using individual patient data (IPD) and external controls can provide clinically relevant estimates of relative treatment effectiveness. We previously reported that DB administered after relapse was associated with longer OS than historical controls who had not received anti-GD2 immunotherapy; however, that framework did not include a cohort of patients who relapsed after first-line DB maintenance and therefore could not address a potential carryover or legacy effect of prior treatment (13).

To address this gap, the present study extends the historical-control framework by incorporating a cohort of patients who relapsed after receiving DB during first-line maintenance. This design enables an adjusted indirect comparison of OS across three clinically relevant populations: patients treated with DB after relapse without prior DB exposure (the N/DB cohort), historical controls with no prior anti-GD2 exposure (the N/N cohort), and patients who relapsed after prior DB maintenance and did not receive subsequent DB (the DB/N cohort). In doing so, the study aims to clarify both the effectiveness of DB in the relapsed setting and whether any survival benefit associated with first-line DB persists after relapse, reflecting a potential legacy or carryover effect of first-line DB immunotherapy.

## 2 Materials and methods

### 2.1 Study design and plan

This study was an unanchored indirect comparison of time-to-event outcomes using IPD on DB (Qarziba^®^) from multiple sources. Naive comparison (unadjusted) and population-adjusted comparison (with population adjustment using inverse probability weighting) were conducted.

Three cohorts were included in the comparison:

- N/N cohort (no DB during first-line therapy → **n**o DB after relapse): historical controls who did not receive anti-GD2 immunotherapy at any stage of treatment (described in detail previously (13));
- DB/N cohort (DB during first-line maintenance → **n**o DB after relapse): patients who relapsed after receiving DB as first-line maintenance and did not receive DB after relapse; and
- N/DB cohort (no DB during first-line therapy → DB after first or subsequent relapse): patients with relapsed neuroblastoma who were treated with DB after relapse but had not previously received DB during first-line therapy.

Harmonized inclusion criteria were applied across all study populations. To ensure analytical consistency, the observed post-relapse index date in the N/DB cohort (defined as the date of the first DB dose after relapse) served as the reference for defining the auxiliary index date in the comparator cohorts. For the N/N and DB/N cohorts, the auxiliary index date (representing the potential date of the first DB dose) was imputed based on the interval between first relapse and initiation of DB observed in the N/DB cohort.

The adjusted comparison used propensity score (PS) reweighting to balance the study populations with respect to key baseline prognostic factors. Differences in OS were analyzed using weighted Kaplan–Meier methods and weighted Cox proportional hazards models.

Prespecified deterministic sensitivity analyses were performed to assess the robustness of the results and conclusions from the base-case analysis. These analyses evaluated the impact of potential uncertainties related to the definition of the index date, baseline patient characteristics, and treatment effects.

### 2.2 Selection of sources

#### 2.2.1 Historical controls (N/N cohort)

The N/N cohort has been described in detail elsewhere (13). Historical control patients were selected from the study by Garaventa et al., 2009 (14) (i.e., patients enrolled in the Italian Neuroblastoma Registry [INBR]) and from the HR-NBL-1/SIOPEN study (restricted to patients randomized during the R1 phase). To maximize comparability between the study and historical control populations, the following eligibility criteria were applied to both patient groups:

- date of initial diagnosis ≥ 1999;
- age at initial diagnosis ≥ 12 months;
- age at first relapse ≥ 12 months;
- International Neuroblastoma Staging System (INSS) stage 4 at initial diagnosis or first relapse that was not local; and
- survival beyond the auxiliary index date (see Section 2.4.1.).

#### 2.2.2 Dinutuximab beta in the treatment of relapsed neuroblastoma (N/DB cohort)

In our previous analysis (13), six studies evaluating DB for the treatment of relapsed neuroblastoma were identified as potential sources for indirect comparison with historical controls: APN311–202 V1+V2 and V3 (17), APN311–304 (18), APN311–303 (17), Wieczorek et al., 2023 (19), APN311–201 (20), and APN311–101 (22). Of these, three studies were included in the present indirect comparison: APN311–202 V1+V2 and V3 (17), APN311–304 (18), and APN311–303 (17) (**Table 1**).

**Table 1:** Identified studies of DB in the treatment of relapsed neuroblastoma.

Study, cohort	Key information	Comment
APN311-202, V1+V2 (stage I) (17)	Prospective, 51 patients with relapsed disease (50 with survival data)	Included in analysis
APN311-202, V3 (stage II) (17)	Prospective, 45 patients with relapsed disease (21 in experimental arm, without IL-2; 44 with survival data)	Included in analysis
APN311–304 (18)	Prospective, 19 patients with relapsed disease OS: long-term follow-up data available (18 patients with relapsed disease)	Included in analysis
APN311-303 (compassionate use) (17, 21),	Retrospective, 30 patients with relapsed disease (17) Compassionate use (treatment or patient selection unknown and/or not representative to whole population)	Included in analysis
Wieczorek et al., 2023 (19)	Only 8 patients with relapsed disease	Excluded from analysis
APN311-201 (Flaadt et al., 2023) (20)	No IPD Inappropriate dosing (28-day cycle) Inappropriate treatment (haplo-SCT required as treatment of all patients)	Excluded from analysis
APN311-101 (Ladenstein et al., 2013) (22)	Inappropriate dosing (28-day cycle; max 3 cycles; only 10 patients with recommended dose) No IPD for relapsed patients (16 refractory)	Excluded from analysis

IL-2 – interleukin-2 (aldesleukin); IPD – individual patient data; OS – overall survival; SCT – stem cell transplantation.

The selection criteria for inclusion in the indirect comparison were as follows:

- patient population: at least 10 patients with relapsed neuroblastoma;
- treatment: DB administered according to the approved schedule and relevant clinical guidelines; and
- availability of IPD.

Patients treated for relapsed disease were identified from the selected studies. Eligible patients had experienced at least one relapse after the initial diagnosis of neuroblastoma and had received treatment for that relapse before initiation of DB immunotherapy. Overall, the study included 142 patients with relapsed neuroblastoma treated with DB, with or without interleukin IL-2, for whom survival data were available.

#### 2.2.3 Relapsed first-line immunotherapy survivors (DB/N cohort)

Among the 370 patients in the full analysis set of the APN311–302 trial (R2 cohort), three were excluded due to missing EFS and/or OS data. Of the remaining 367 patients with available survival data, 146 experienced an EFS event. Eight patients who died before relapse were excluded, leaving 138 patients in the DB/N cohort. No secondary malignancies were observed as EFS events. However, one patient subsequently developed another malignancy. The remaining 138 patients were assessed using eligibility criteria consistent with those applied to the historical controls (13):

- date of initial diagnosis ≥ 1999: all patients met this criterion, with diagnoses occurring between 2007 and 2014;
- age at initial diagnosis ≥ 12 months: nine patients did not meet this criterion;
- age at first relapse ≥ 12 months: all patients met this criterion;
- INSS stage 4 at initial diagnosis or first relapse that was not local: 26 patients did not meet this criterion, including 12 with INSS< 4 and 20 with a local first relapse; and
- survival beyond the auxiliary index date (see Section 2.4.1.).

Overall, 33 patients were excluded, leaving 105 patients in the DB/N cohort. This cohort included 39 patients whose relapse occurred within 25 days after the last DB dose.

#### 2.2.4 Summary of patient disposition

The study included 142 patients with relapsed neuroblastoma treated with DB, with or without IL-2 (N/DB cohort), 105 patients who relapsed after first-line DB maintenance immunotherapy (DB/N cohort), and 82 historical controls with relapsed neuroblastoma and no prior anti-GD2 immunotherapy (N/N cohort). Overall, 329 patients with relapsed neuroblastoma were assessed (**Table 2**, **Figure 1**).

**Table 2:** Patient disposition – relapsed neuroblastoma.

Study	Arm	Cohort	N	%, cohort	%, total sample
INBR control	Historical control	N/N	34	41.5%	10.3%
R1 control	Historical control	N/N	48	58.5%	14.6%
Totals		N/N	82	100.0%	24.9%
APN311-303	DB in R/R	N/DB	30	21.1%	9.1%
APN311-202, V1+V2	DB in R/R	N/DB	50	35.2%	15.2%
APN311-202, V3	DB in R/R	N/DB	44	31.0%	13.4%
APN311-304	DB in R/R	N/DB	18	12.7%	5.5%
Totals		N/DB	142	100.0%	43.2%
APN311–302 R2	DB in FL	DB/N	105	100%	31.9%
Totals		DB/N	105	100.0%	31.9%

N/N - no DB in first-line →no DB after relapse cohort; N/DB - no DB →DB cohort; DB/N - DB →no DB cohort.

**Figure 1:**
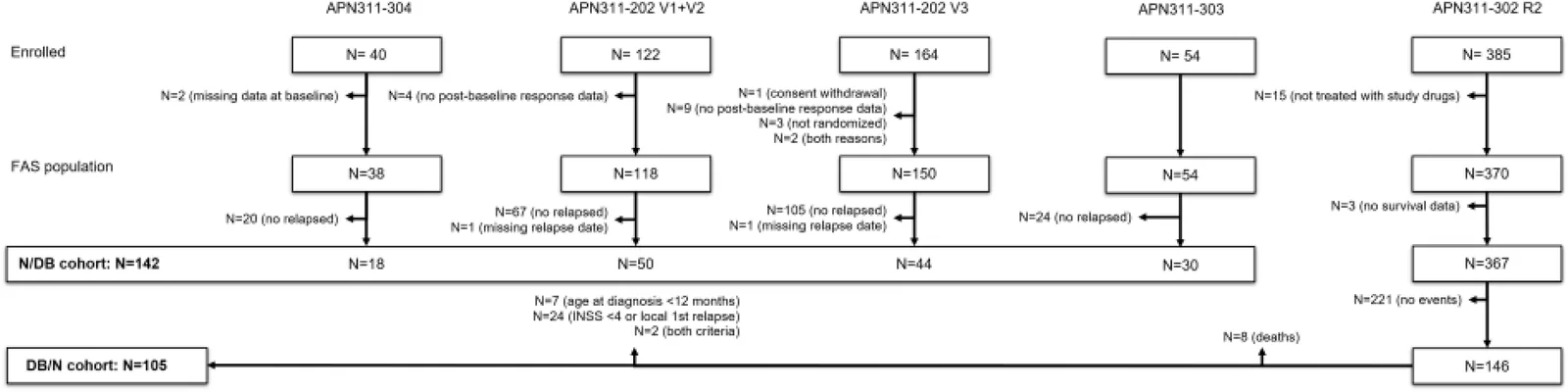
Patient disposition from DB trials.

### 2.3 Data management

Similar to our previous study, the information extracted for each patient was determined by the availability of corresponding data for the historical controls and included the following:

1). Patient characteristics:

- date of birth^1^;
- sex;
- date of initial diagnosis;
- date of first relapse;
- date of immunotherapy initiation (first administration of DB) – the N/DB cohort only;
- MYCN status (amplified, non-amplified, or missing);
- INSS stage at initial diagnosis (stage or missing).

2). Outcomes (OS):

- censoring date (date of the last follow-up examination or last contact);
- date of death;
- event classifier (event, 1; no event – censored, 0).

### 2.4 Statistical analysis

#### 2.4.1 Definition of the post-relapse index date

For the DB cohorts, the origin for OS analyses was defined as the initiation of DB treatment (i.e., the date of the first DB dose, regardless of concomitant IL-2 treatment). Because patients in the N/N and DB/N cohorts did not receive DB after relapse, an auxiliary index date was estimated based on the date of first relapse.

In all trials evaluating DB treatment for relapsed neuroblastoma (N/DB), patients had experienced at least one relapse or disease progression before enrollment. For the historical control group (N/N) and for patients who relapsed after DB immunotherapy (DB/N), the analysis was anchored to the first relapse event. Consequently, only patients enrolled in the DB trials at their first relapse were included in the estimation of the auxiliary index date. Because information on the number of previous relapses or disease progressions was unavailable for 30 patients in the APN311–303 trial, data from patients enrolled in the other trials were used to inform this analysis (112 patients, including 75 at first relapse).

The utility of ordinary least squares regression, generalized linear models, parametric survival regression, and median quantile regression for predicting the auxiliary index date among patients in the N/N and DB/N cohorts was evaluated. Model performance was assessed using internal predictive performance metrics, including the mean absolute error and root mean squared error. Based on these measures, the median quantile regression model demonstrated the best predictive performance and was selected for the analysis (**Supplementary Table 1**).

After defining the auxiliary index date for each patient, the final eligibility criterion was applied. Sixty-three patients were excluded because their OS follow-up ended before the index date (i.e., they died or were censored between the date of first relapse and the index date). This restriction prevented negative time at risk and reduced immortal time bias in the post-landmark analyses. The final patient disposition is shown in **Figure 2**. In total, 266 patients were included in the PS-weighted analyses.

**Figure 2:**
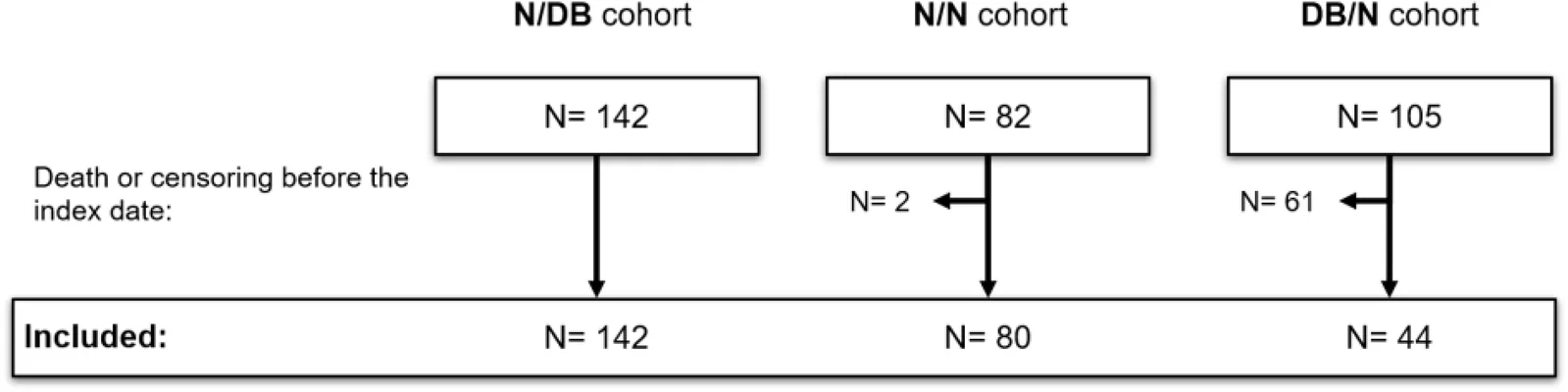
Final patient disposition.

#### 2.4.2 Comparison of patient characteristics

Categorical variables were compared between cohorts using the χ^2^ test. Continuous variables were compared using the Kruskal–Wallis H test or, for PS-weighted cohorts, a test of the linear regression coefficient for the cohort indicator.

#### 2.4.3 Adjusted comparison of overall survival

A population-adjusted comparison of OS was performed using inverse probability weighting. Propensity score reweighting was applied to balance the study cohorts with respect to key baseline prognostic factors. Multinomial PSs were estimated for the three-level cohort variable (N/N, DB/N, and N/DB) using multinomial logistic regression with covariates measured at the relapse preceding the observed (N/DB) or imputed (N/N, DB/N) index date. The following baseline prognostic factors, selected based on their availability in the historical control cohort (13), were considered in the PS model:

- time (years) from diagnosis to the most recent relapse;
- age at the recent relapse;
- sex (male, female);
- MYCN status (amplified, non-amplified, or missing);
- INSS stage (≥ 4 vs other).

Model selection and assessment were based on the Box-Cox test, the generalized Hosmer-Lemeshow test, and the log-likelihood. Interactions terms and quadratic terms for continuous covariates were considered to account for potential effect modification and nonlinearity, respectively, and were retained if they substantially improved model fit. Candidate interaction and quadratic terms were evaluated using likelihood-ratio (LR) tests for nested models (two-sided α=0.05). Model building followed a forward, hierarchically well-formulated selection strategy. Among candidate terms with p<0.05, the term yielding the largest increase in the LR χ^2^ statistic was added first. The model was then refitted, and the remaining candidate terms were re-evaluated in descending order of LR χ^2^. Each newly added term was retained only if it continued to improve model fit (LR test, p<0.05); otherwise, it was removed.

The PS assessment was performed with the N/DB cohort as the reference group by estimating the probability of inclusion in this cohort (**Supplementary Table 2**). To compare all cohorts using the covariate distribution of the N/DB cohort as the target population, average treatment effect in the treated weights were constructed. Letting $e_{cohort}\left(X\right)=\text{Pr}\left(\text{cohort}|X\right)$, the weights were defined as *w* = 1 for patients in the N/DB cohort and $w=e_{N/DB}\left(X\right)/e_{cohort}\left(X\right)$ for patients in the N/N, and DB/N cohorts. Extreme weights (1st–99th percentiles) were trimmed to improve stability and mitigate near-violations of the positivity assumption. Visual overlap of the $e_{cohort}\left(X\right)$ densities across cohorts was also evaluated, together with the distribution of weights and the effective sample size as additional diagnostic measures (15, 16).

The PS-weighted IPD were analyzed using a weighted Cox proportional hazards model with robust variance estimation and the weighted Kaplan-Meier method. A doubly robust weighted Cox model was evaluated in sensitivity analyses. The following covariates were included in the doubly robust model: i) time (years) from diagnosis to the most recent relapse; ii) age at the most recent relapse; iii) sex; iv) MYCN status; and v) INSS stage.

#### 2.4.4 Missing data and statistical software

For categorical variables, missing values were handled by introducing an explicit “missing” category. No adjustment was performed for missing continuous variables. Patients with missing survival data were excluded from the analysis, as described in Section 2.2.

Median follow-up time from the observed or imputed index date was calculated using reverse Kaplan-Meier method with 95% CI for weighted estimates calculated with bootstrap method. Kish effective sample size was provided for weighted estimates.

A p-value of less than 0.05 was considered significant. Statistical analyses were performed using StataNow 19.5SE (StataCorp., College Station, TX, USA).

## 3 Results

### 3.1 Patient characteristics

Before reweighting, significant differences were observed between the N/N, DB/N, and N/DB cohorts across all assessed baseline characteristics except sex (p=0.300). These characteristics included age at relapse, year of diagnosis, time from diagnosis to the index date, MYCN status, and INSS stage at diagnosis (all p<0.001). After PS reweighting, no significant differences were observed among the N/N, DB/N and N/DB cohorts across the assessed baseline characteristics, except for year of diagnosis and INSS stage at diagnosis (both p<0.001; **Table 3**).

**Table 3:** Characteristics of the study population by cohort.

		Characteristics before PS reweighting				Characteristics after PS reweighting^A^			
		N/N cohort (N = 80)	DB/N cohort (N = 44)	N/DB cohort (N = 142)	P-value	N/N cohort (Σw=105.2)	DB/N cohort (Σw=103.4)	N/DB cohort (Σw=142)	P-value
Age at the relapse, years	Median (IQR)	5.87 (4.51 to 6.98)	4.79 (3.35 to 6.77)	6.70 (4.59 to 9.69)	**<0.001**	6.22 (5.01 to 7.93)	6.75 (4.25 to 7.69)	6.70 (4.59 to 9.70)	0.150
	Mean (SD or SE)	6.27 (2.80)	5.39 (3.08)	7.42 (4.17)		6.82 (0.44)	6.45 (0.36)	7.42 (0.35)	
Year of diagnosis	Median (IQR)	2004 (2002 to 2006)	2011 (2010 to 2012)	2009 (2008 to 2012)	**By design (<0.001)**	2004 (2002 to 2006)	2011 (2010 to 2012)	2009 (2008 to 2012)	**By design (<0.001)**
	Mean (SD or SE)	2004.01 (2.76)	2010.89 (1.13)	2009.52 (3.38)		2004.03 (0.36)	2010.82 (0.15)	2009.52 (0.28)	
Time from diagnosis, years	Median (IQR)	1.67 (1.28 to 2.17)	1.43 (1.12 to 2.06)	2.08 (1.29 to 3.94)	**<0.001**	1.91 (1.51 to 3.64)	2.32 (1.45 to 3.27)	2.09 (1.29 to 3.94)	0.239
	Mean (SD or SE)	2.08 (1.30)	1.72 (1.02)	2.97 (2.58)		2.58 (0.24)	2.48 (0.20)	2.97 (0.22)	
Sex, n (%)	Male	53 (66.2%)	23 (52.3%)	81 (57.0%)	0.248	54.8 (52.1%)	54.7 (52.9%)	81 (57.0%)	0.815
	Female	27 (33.8%)	21 (47.7%)	61 (43.0%)		50.4 (47.9%)	48.7 (47.1%)	61 (43.0%)	
MYCN status, n (%)	not amplified	52 (65%)	35 (79.6%)	115 (81.0%)	**0.044**	81.5 (77.4%)	82.7 (80.0%)	115 (81.0%)	0.912
	amplified	23 (28.8%)	6 (13.6%)	18 (12.7%)		17.3 (16.4%)	12.1 (11.7%)	18 (12.7%)	
	missing	5 (6.2%)	3 (6.8%)	9 (6.3%)		6.5 (6.1%)	8.6 (8.3%)	9 (6.3%)	
INSS stage at diagnosis, n (%)	4 or 4S	78 (97.5%)	44 (100%)	111 (78.2%)	**<0.001**	102.2 (97.1%)	103.4 (100.0%)	111 (78.2%)	**<0.001**
	Others^B^	2 (2.5%)	0 (0%)	31 (21.8%)		3.0 (2.9%)	0.0 (0.0%)	31 (21.8%)	

^A^ Σw – sum of weights.

^B^ combined due to the unequal distribution of each stage between arms (1, 2A, 2B, 3, missing).

IQR, interquartile range; INSS, International Neuroblastoma Staging System; N/N - no DB in first-line →no DB after relapse cohort; N/DB - no DB →DB cohort; DB/N - DB →no DB cohort; SD, standard deviation; SE, standard error; PS, propensity score.

Bold values indicate statistically significant results.

Median follow-up time differed across cohorts. In the unweighted analyses, median follow-up was 4.68 years (95% CI, 3.77–6.42) in the N/N cohort (N = 80), 1.72 years (95% CI, 1.26–2.26) in the DB/N cohort (N = 44), and 4.20 years (95% CI, 3.84–4.70) in the N/DB cohort (N = 142). After weighting, the corresponding medians were 6.17 years (95% CI, 4.20–8.35; effective sample size of 58.51), 1.40 years (95% CI, 0.86–2.19; effective sample size of 37.91), and 4.20 years (95% CI, 3.90–4.70; effective sample size of 142), respectively.

### 3.2 Base-case analysis

There was no evidence of a legacy effect of first-line maintenance immunotherapy with DB after the first relapse. Among patients relapsed after first-line maintenance immunotherapy with DB, post-landmark OS, with the auxiliary index date corresponding to the first DB dose after relapse, did not differ from that of the historical control cohort (hazard ratio [HR] = 1.26, 95% CI 0.70–2.26; p=0.437). As previously reported (13), patients treated with DB after relapse had significantly better OS than the historical control cohort (HR = 0.53, 95% CI 0.35–0.81; p=0.003). Similarly, patients treated with DB after relapse had significantly better OS than patients who relapsed after first-line DB maintenance immunotherapy (HR = 0.42, 95% CI 0.25–0.72; p=0.001). The results of the base-case analysis are presented in **Table 4** and **Figure 3**.

**Table 4:** Results of weighted Cox proportional hazards model.

Cohort/comparison	HR (95% CI)	P value
N/N (historical controls)	*reference*	–
DB/N vs N/N	1.26 (0.70 to 2.26)	0.437
N/DB vs N/N	0.53 (0.35 to 0.81)	**0.003**
Other comparisons		
N/DB vs DB/N	0.42 (0.25 to 0.72)	**0.001**

CI, confidence interval; HR, hazard ratio; N/N - no DB in first-line →no DB after relapse cohort; N/DB - no DB →DB cohort; DB/N - DB →no DB cohort. Bold values indicate statistically significant results.

**Figure 3:**
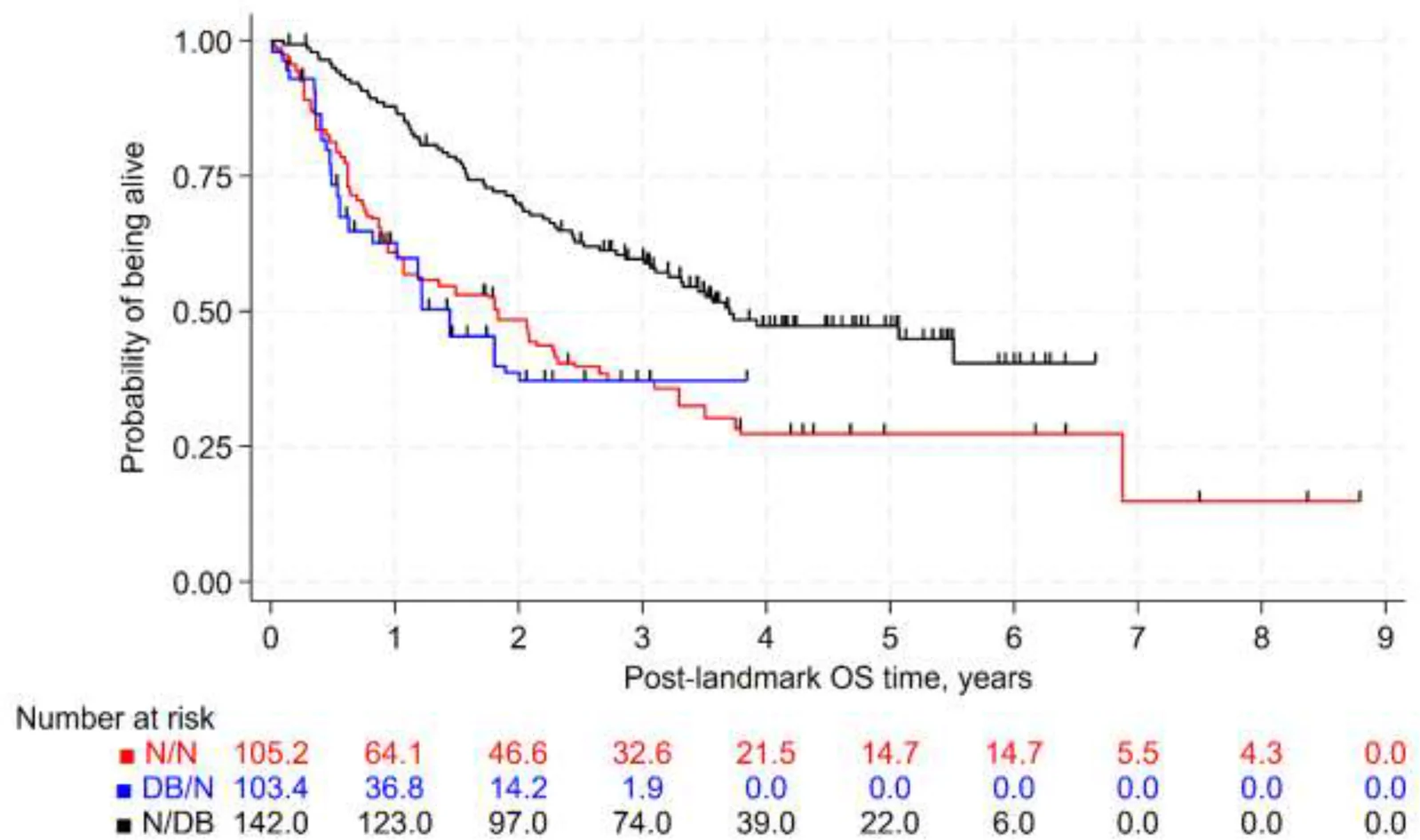
Weighted Kaplan-Meier plot of overall survival (OS) from the index date (DB initiation in the N/DB cohort).

### 3.3 Sensitivity analyses

Sensitivity analyses across alternative scenarios and subgroups supported the robustness of the base-case findings (**Supplementary Table 3**). The overall conclusions remained unchanged across all sensitivity analyses, including the use of a doubly robust model; unweighted Cox proportional hazards models with or without covariate adjustment; exclusion of patients who relapsed during first-line DB maintenance immunotherapy, defined as relapse within 25 days after the last DB dose, from the DB/N cohort; exclusion of IL-2-treated patients from the N/DB cohort; an auxiliary index date derived from time since diagnosis; and restriction of the historical control cohort to patients enrolled in the HR-NBL-1/SIOPEN study. The only notable exception was the analysis excluding IL-2-treated patients from the N/DB cohort, in which the reduced sample size and lower number of events resulted in loss of statistical significance for the OS difference between the N/DB cohort and the comparator cohorts. However, the direction of the association was consistent with that observed in the base-case analysis.

## 4 Discussion

The present analysis extends the previously published indirect comparison of DB versus historical controls by incorporating a clinically distinct cohort of patients who relapsed after first-line DB maintenance immunotherapy (13). Accordingly, the study addressed two complementary objectives: to evaluate the association between DB administered after relapse and OS, and to assess whether first-line DB maintenance was associated with a persistent post-relapse survival advantage when DB was not reintroduced. The latter objective represents the novel extension of the previously published framework, whereas the comparison of DB after relapse with historical controls reassesses the association reported previously (13). Consistent with the earlier analysis, DB treatment after relapse was associated with longer OS than in DB-unexposed historical controls (13).

This finding is clinically important because the benefit of anti-GD2 immunotherapy in the first-line setting is well established, whereas it has remained unclear whether this benefit extends to improved survival after relapse. Our findings do not support the presence of a persistent post-relapse survival advantage among patients previously exposed to DB. Instead, they suggest that the observed survival benefit is primarily associated with the use of DB in the relapsed setting. This distinction is clinically relevant because it suggests that post-relapse prognosis may be influenced more by the biological characteristics of the relapsed disease and the effectiveness of salvage treatment than by prior maintenance exposure alone (23).

At first glance, the absence of a legacy effect may appear counterintuitive given the well-established efficacy of anti-GD2 immunotherapy in the first-line setting. However, this apparent discrepancy is biologically and clinically plausible. Although first-line DB may improve EFS and OS by delaying relapse or reducing the risk of recurrence, once relapse occurs the disease may represent a biologically selected, treatment-resistant population in which post-relapse outcomes are determined primarily by factors such as the timing of relapse, disease burden, site of recurrence, and response to salvage therapy (23–25). Another factor that may influence outcomes after anti-GD2 retreatment is the potential development of treatment resistance. Preclinical and translational studies suggest that approximately 40–50% of patients exhibit primary or acquired resistance to anti-GD2–based therapies (26, 27). However, clinical observations indicate that responses may still occur after prior anti-GD2 exposure, suggesting that resistance is heterogeneous and may increase with successive lines of therapy. Further studies are warranted to clarify the mechanisms underlying resistance and their clinical relevance (12).

Particularly relevant to the interpretation of our findings is the role of prior anti-GD2 exposure. In a retrospective two-center case series, Wieczorek et al. (6) reported encouraging activity of DB combined with chemotherapy, even in heavily pretreated patients, including those previously exposed to DB during first-line therapy. Among the 14 patients with prior DB exposure, the objective response rate was 71%. Although that study was small and noncomparative, it suggested that prior anti-GD2 exposure does not necessarily preclude subsequent clinical responsiveness to GD2-directed therapy. Similarly, a French study found that prior anti-GD2 immunotherapy did not appear to affect the efficacy of DB chemo-immunotherapy at disease progression (8). Our findings are consistent with these observations but also extend them. Rather than providing a direct estimate of the efficacy of DB retreatment, our results suggest that prior first-line DB maintenance immunotherapy alone is not associated with improved post-relapse survival in the absence of DB reintroduction after relapse (6, 13). The N/DB cohort did not uniformly receive DB alone: DB was administered with or without IL-2, without concomitant cytotoxic chemotherapy, after relapse-directed treatment had achieved disease control. It therefore mainly represents consolidation/maintenance rather than contemporary chemoimmunotherapy-based reinduction of active disease. Randomized BEACON Immuno and observational studies support DB combined with chemotherapy during reinduction, including activity in some patients previously exposed to anti-GD2 therapy (6, 8, 9, 12). Other GD2-directed options, including GD2-CART01, DB following haploidentical HSCT, and naxitamab plus GM-CSF, have also shown activity in selected relapsed/refractory populations (10, 20, 28). However, differences in disease status, treatment line, and prior therapy preclude direct comparison with the present OS analysis.

In our study, patients in the N/DB cohort differed from those in the comparator cohorts with respect to INSS stage at diagnosis and may also have had more heavily pretreated disease at the time of relapse, as some patients may have experienced multiple relapses before the DB-treated relapse included in the analysis. In contrast, the N/N and DB/N cohorts were restricted to patients with INSS stage 4 or 4S, and follow-up was initiated after the first relapse. Although the difference in INSS stage at diagnosis could theoretically have favored longer OS in the N/DB cohort, the greater post-relapse disease burden and more extensive treatment history in this cohort would be expected to offset, and potentially outweigh, this source of bias.

This study has several limitations that should be considered when interpreting the findings. First, although the historical control cohorts comprised patients who had not received anti-GD2 immunotherapy within the data sources used for this comparison, it cannot be excluded that some patients may have received an anti-GD2 agent through other clinical studies or treatment pathways not captured in the available datasets.

Second, the historical control datasets contained only a limited number of baseline and disease-related variables, which restricted the extent to which differences between cohorts could be addressed statistically. Although the adjusted analyses incorporated the key prognostic factors consistently available across all cohorts, residual confounding cannot be excluded because information on important clinical characteristics, including prior treatment intensity, the number of previous relapses or disease progressions, disease burden at relapse, and post-relapse subsequent therapies, was unavailable and therefore could not be harmonized across datasets. Consequently, despite the use of PS reweighting and doubly robust modeling, complete exchangeability between cohorts cannot be assumed.

Third, temporal differences between the study cohorts should be considered when interpreting the findings. Nearly half of the DB-treated patients were diagnosed after 2009, whereas the historical control cohort comprised patients diagnosed between 1999 and 2009. Advances in supportive care, diagnostic methods, salvage therapy, risk stratification, and access to specialized treatment over time may have contributed, at least in part, to the more favorable OS observed in the DB-treated cohort, independent of the direct effect of DB. However, this concern is partially mitigated by the comparison with the DB/N cohort, which represents a more contemporary population that received first-line DB maintenance immunotherapy. Therefore, no evidence was found to suggest that differences in calendar period alone accounted for the survival advantage associated with DB treatment after relapse.

The N/DB cohort predominantly comprised patients whose relapse had responded to salvage therapy and whose disease was controlled before DB initiation, whereas disease status at the index date was unavailable for the N/N and DB/N cohorts. This may have introduced residual confounding in favor of N/DB if active or progressive disease was more frequent in the comparator cohorts. However, most screened survivors of salvage therapy were eligible for DB immunotherapy: 122 of 124 patients (98.4%) in APN311–202 V1+V2, 163 of 166 (98.2%) in APN311–202 V3, and 40 of 46 (87.0%) in APN311-304. Thus, failure to achieve sufficient disease control for DB initiation appeared uncommon, although eligibility does not demonstrate comparable distributions of response depth, residual disease, or occult progression across cohorts. Moreover, in the DB/N cohort, only one of 44 patients had documented progression or relapse, or started a new treatment regimen, within 30 days after the imputed index date, and no event occurred during the first 7 days. Overt progressive disease at the index date was therefore unlikely to have been frequent in DB/N cohort, although subclinical progression cannot be excluded; no comparable information was available for N/N. Disease response and tumor burden could not be included in the propensity-score model because they were not measured consistently. Adjustment for the interval from relapse to the observed or imputed index date aligned survival to the potential start of DB but could not correct for potential differences in disease control. Residual confounding therefore remains, particularly for the N/DB versus N/N comparison.

An additional source of heterogeneity arises from differences between the two historical control datasets (13). In the R1 control cohort, only patients who relapsed after achieving an initial complete response (CR) were available for analysis, whereas the INBR cohort also included patients who had achieved a prior partial response (PR). By contrast, the DB studies included patients with either a previous CR or PR. Because relapse after a PR is generally associated with a poorer prognosis than relapse after CR, the inclusion of the R1 cohort in the pooled historical control group may have shifted the prognosis of the control population in a more favorable direction. If so, this would likely have resulted in an underestimation, rather than an overestimation, of the survival benefit associated with DB.

Finally, differences in relapse history across the cohorts should be acknowledged. Only patients at first relapse were included in the historical control (N/N) and post-first-line DB maintenance relapse (DB/N) cohorts, whereas the DB-treated cohort (N/DB) also included patients who may have experienced more than one relapse or disease progressions before initiation of DB treatment. This asymmetry in cohort composition introduces the potential for bias because patients with multiple relapses generally have a less favorable prognosis. We sought to partially address this limitation by including time from diagnosis to the index date in the adjustment model, thereby accounting, at least indirectly, for differences in disease trajectory. Importantly, the sensitivity analysis using an auxiliary index date derived from time since diagnosis, rather than time from the most recent relapse, yielded results consistent with the base-case analysis, supporting the robustness of the main conclusions despite this structural limitation.

Taken together, these limitations reflect the inherent challenges of conducting indirect comparative effectiveness research in relapsed neuroblastoma, particularly in a setting where randomized placebo-controlled trials are not feasible. Consequently, residual confounding cannot be excluded. As in our previous study (13), the adjusted analyses accounted for the principal post-relapse prognostic factors consistently available across the study cohorts. However, other clinically relevant variables, including end-of-induction response, detailed histologic characteristics, anaplastic lymphoma kinase status, transplant type (tandem transplantation was not performed during the study period), the number of first-line anti-GD2 treatment cycles, and the chemotherapy backbone used at relapse, were not available in a harmonized form across datasets. Although robust evidence that several of these variables independently influence OS after relapse, beyond established prognostic factors such as time to relapse, age, INSS stage, and MYCN, is limited, some degree of residual confounding is likely.

This study has several important strengths. Harmonized eligibility criteria were applied across all cohorts, followed by statistical adjustment for the baseline prognostic characteristics consistently available across the datasets, thereby improving clinical comparability and reducing the risk of confounding inherent in indirect comparisons. The analysis integrated all available IPD from eligible DB studies with data from two independent historical control cohorts (13), providing a comparatively large dataset for a rare and heterogeneous disease. The study also addresses a clinically relevant question in a setting where a contemporaneous randomized comparison of DB versus placebo or no immunotherapy after relapse would be ethically difficult to justify. Finally, the consistency of the base-case findings across prespecified sensitivity analyses supports the robustness and methodological rigor of the results despite the observational nature of the comparison.

The DB/N cohort comprised patients who relapsed after first-line DB maintenance but did not receive DB after relapse; therefore, the present study evaluated only the existence of a potential legacy effect. However, because the N/DB cohort had better survival than both the historical control and DB/N cohorts, and because previous studies (6, 8, 10, 12). suggest that DB-containing chemoimmunotherapy retains clinical activity in some patients with prior exposure to anti-GD2 therapy, our findings support continued investigation of DB-based retreatment strategies rather than the assumption that previous first-line DB exposure alone is associated with improved post-relapse prognosis.

Importantly, our findings should not be interpreted as direct evidence of the efficacy of DB retreatment after prior first-line DB. Nevertheless, the consistency of the results across multiple analytical approaches, weighting strategies, and sensitivity analyses strengthens confidence in the overall direction and magnitude of the observed association.

## 5 Conclusions

In this adjusted indirect comparison, DB administered after relapse was associated with significantly longer OS than that observed in both historical controls without prior anti-GD2 exposure and patients who relapsed after first-line DB maintenance immunotherapy and did not receive DB after relapse. No evidence of a legacy effect of first-line DB maintenance immunotherapy on post-relapse survival was found. These findings support DB as a clinically relevant treatment option for relapsed neuroblastoma while highlighting the need for further research on retreatment strategies and treatment sequencing in patients previously exposed to anti-GD2 immunotherapy. However, residual confounding cannot be excluded; therefore, the findings should be regarded as hypothesis-generating and should not be interpreted as establishing causal effects.

## Data Availability

The original contributions presented in the study are included in the article/**Supplementary Material**. Further inquiries can be directed to the corresponding authors.

## Footnotes

1 For patients from the APN311-304 trial, only the year of birth was available; a birthday of 30 June was assumed for every patient

## Supplementary material

Supplementary Table 1 Model for the index date.

Supplementary Table 2 Model for PS weights.

Supplementary Table 3 Sensitivity analyses.
