# Supplementary material for: Overall survival after relapse in high-risk neuroblastoma: an adjusted indirect comparison of post-relapse dinutuximab beta, historical controls, and relapse after first-line dinutuximab beta maintenance
Source: Front Oncol. 2026 Oct 7;16:1933438. doi: 10.3389/fonc.2026.1933438 (PMC13683140; doi:10.3389/fonc.2026.1933438)
Supplement: Supplementary file 1 [file DataSheet1.pdf]

Supplementary Material

SUPPLEMENTARY TABLE 1. Model for the index date.

```
Median regression                                    Number of obs =        75
  Raw sum of deviations 12.67476 (about 5.8550719)
  Min sum of deviations 12.29775                     Pseudo R2     =    0.0297
```

ln_Ts	Coefficient	Std. err.	t	P>|t|	[95% conf.	interval]
age_at_relapse	-.011777	.0309566	-0.38	0.705	-.0735337	.0499798
year_from_diag	.032145	.0416189	0.77	0.443	-.0508825	.1151725
sex						
male	0	(base)				
female	-.0168104	.1487979	-0.11	0.910	-.3136541	.2800332
inss2						
other	0	(base)				
4+	-.1343541	.1837877	-0.73	0.467	-.5010004	.2322922
mycn						
not amplified	0	(base)				
amplified	-.1196964	.208431	-0.57	0.568	-.5355048	.296112
_cons	5.979611	.2103978	28.42	0.000	5.559879	6.399343

SUPPLEMENTARY TABLE 2. Model for PS weights.

```
Multinomial logistic regression                         Number of obs =    266
                                                        LR chi2(18)   =  70.72
                                                        Prob > chi2   = 0.0000
Log likelihood = -229.05784                             Pseudo R2     = 0.1337
```

cohort	Coefficient	Std. err.	z	P>|z|	[95% conf.	interval]
**NN**						
age_at_relapse	.0755123	.0639114	1.18	0.237	-.0497518	.2007763
year_from_diag	3.686447	3.181371	1.16	0.247	-2.548926	9.92182
sex						
female	-.4172984	.324411	-1.29	0.198	-1.053132	.2185355
inss2						
4+	22.71532	18.31001	1.24	0.215	-13.17165	58.60229
mycn						
amplified	8.970673	9.146294	0.98	0.327	-8.955733	26.89708
missing	-8.586537	1676.809	-0.01	0.996	-3295.072	3277.899
mycn#inss2						
amplified#4+	-8.139965	9.14998	-0.89	0.374	-26.0736	9.793665
missing#4+	8.793726	1676.809	0.01	0.996	-3277.692	3295.28
inss2#c.year_from_diag						
4+	-4.067536	3.188781	-1.28	0.202	-10.31743	2.182361
_cons	-22.6505	18.33086	-1.24	0.217	-58.57833	13.27734
**QN**						
age_at_relapse	.0679481	.0727478	0.93	0.350	-.0746349	.2105312
year_from_diag	.0977346	505.8543	0.00	1.000	-991.3585	991.554
sex						
female	.1713919	.3719175	0.46	0.645	-.5575531	.9003368
inss2						
4+	17.43993	1716.756	0.01	0.992	-3347.341	3382.22
mycn						
amplified	.8901164	2736.738	0.00	1.000	-5363.018	5364.798
missing	-.315686	3150.663	-0.00	1.000	-6175.502	6174.871
mycn#inss2						
amplified#4+	-.9299061	2736.738	-0.00	1.000	-5364.838	5362.978
missing#4+	.3973612	3150.663	0.00	1.000	-6174.79	6175.584
inss2#c.year_from_diag						
4+	-.4867973	505.8543	-0.00	0.999	-991.9431	990.9695
_cons	-17.9328	1716.756	-0.01	0.992	-3382.713	3346.848
**NQ**	(base outcome)					

NN - no DB in fist-line → no DB after relapse cohort; NQ - no DB → DB cohort; QN - DB → no DB cohort

SUPPLEMENTARY TABLE 3. *Sensitivity analyses. NN - no DB in fist-line no DB after relapse cohort; NQ - no DB DB cohort; QN - DB no DB cohort.*

	Kaplan-Meier plot	Cox model

0) Base-case analysis

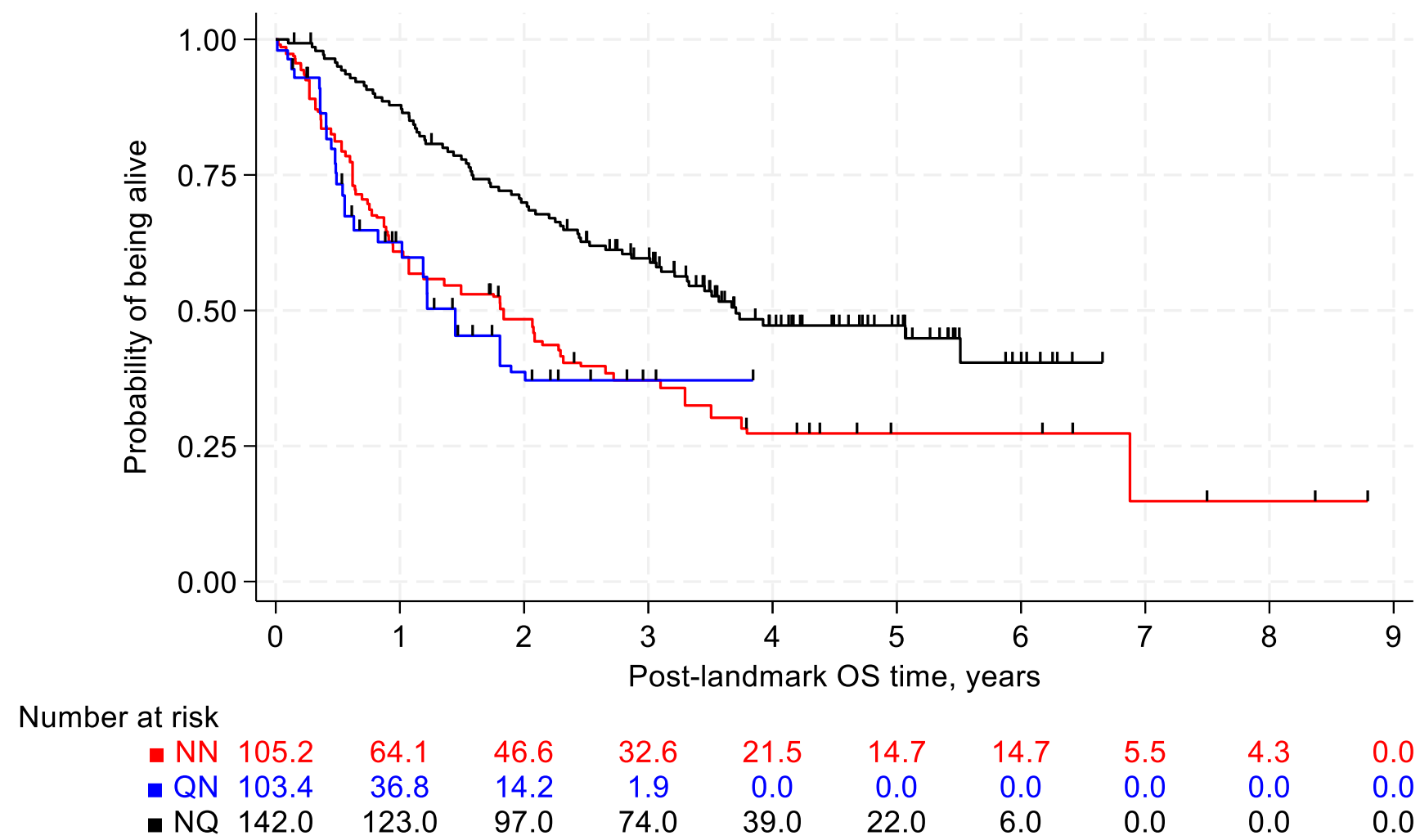

	HR (95% CI)	p Value
**QN vs NN**	1.26 (0.70 to 2.26)	0.437
**NQ vs NN**	**0.53** (0.35 to 0.81)	**0.003**

1) Doubly robust Cox model (Cox model with covariates used for the estimation of the PS weights)

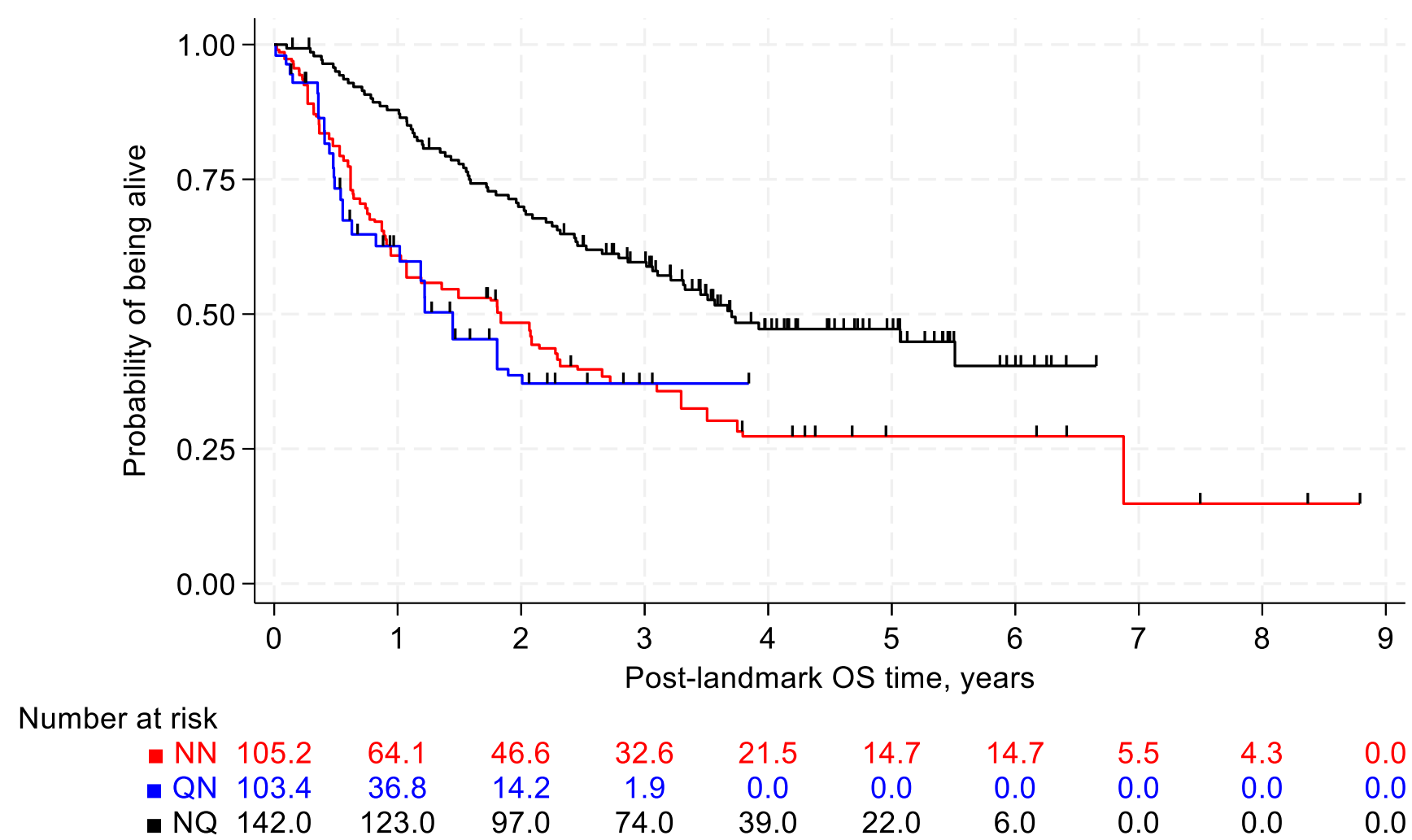

	HR (95% CI)	p Value
**QN vs NN**	1.24 (0.70 to 2.20)	0.454
**NQ vs NN**	**0.57** (0.37 to 0.86)	**0.007**

	Kaplan-Meier plot	Cox model
**2) Unadjusted comparison (no weights)**	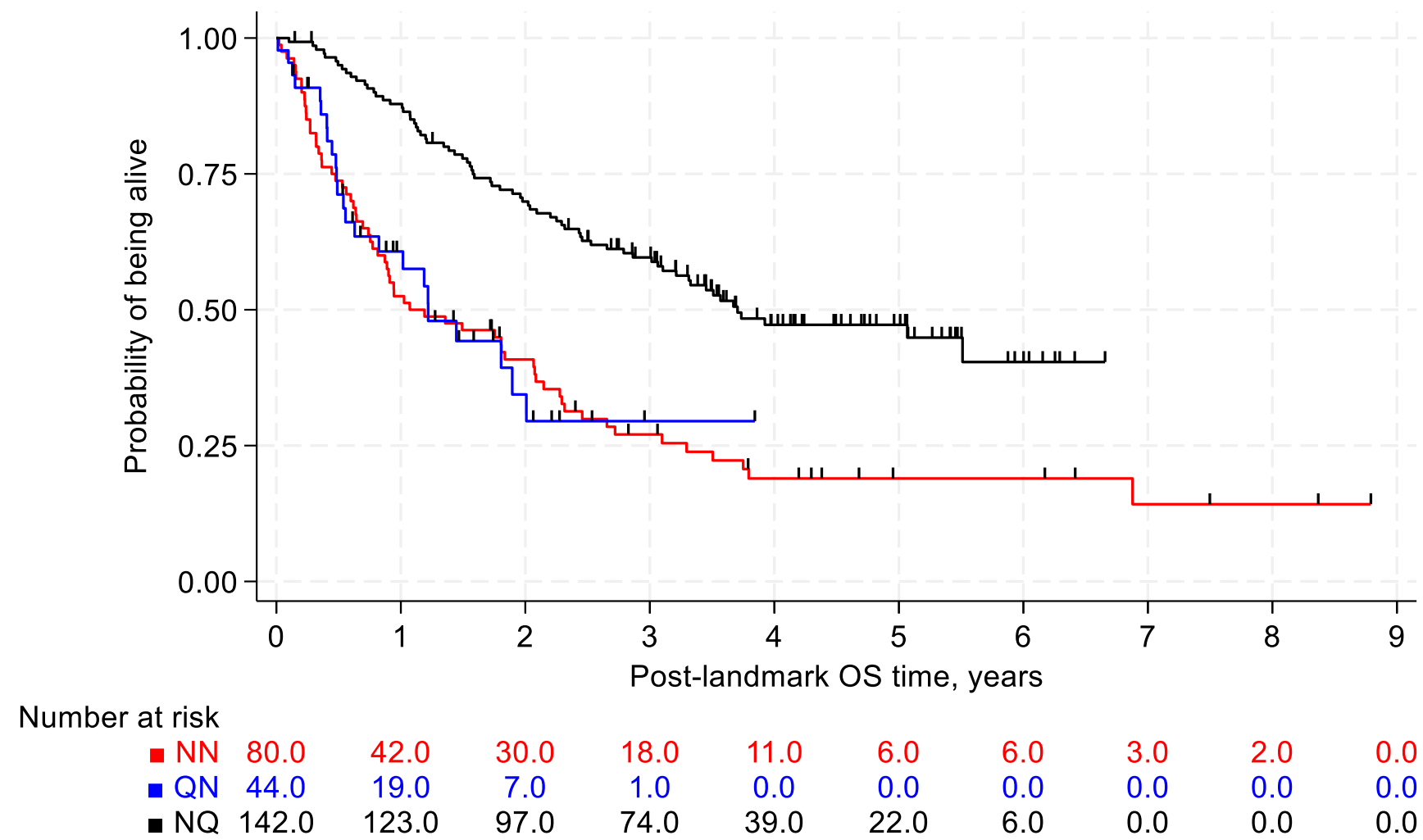	see below
**3) QN cohort: patients with a relapse during 1st line DB maintenance (i.e., within a period of 25 days after the last DB dose) were excluded**	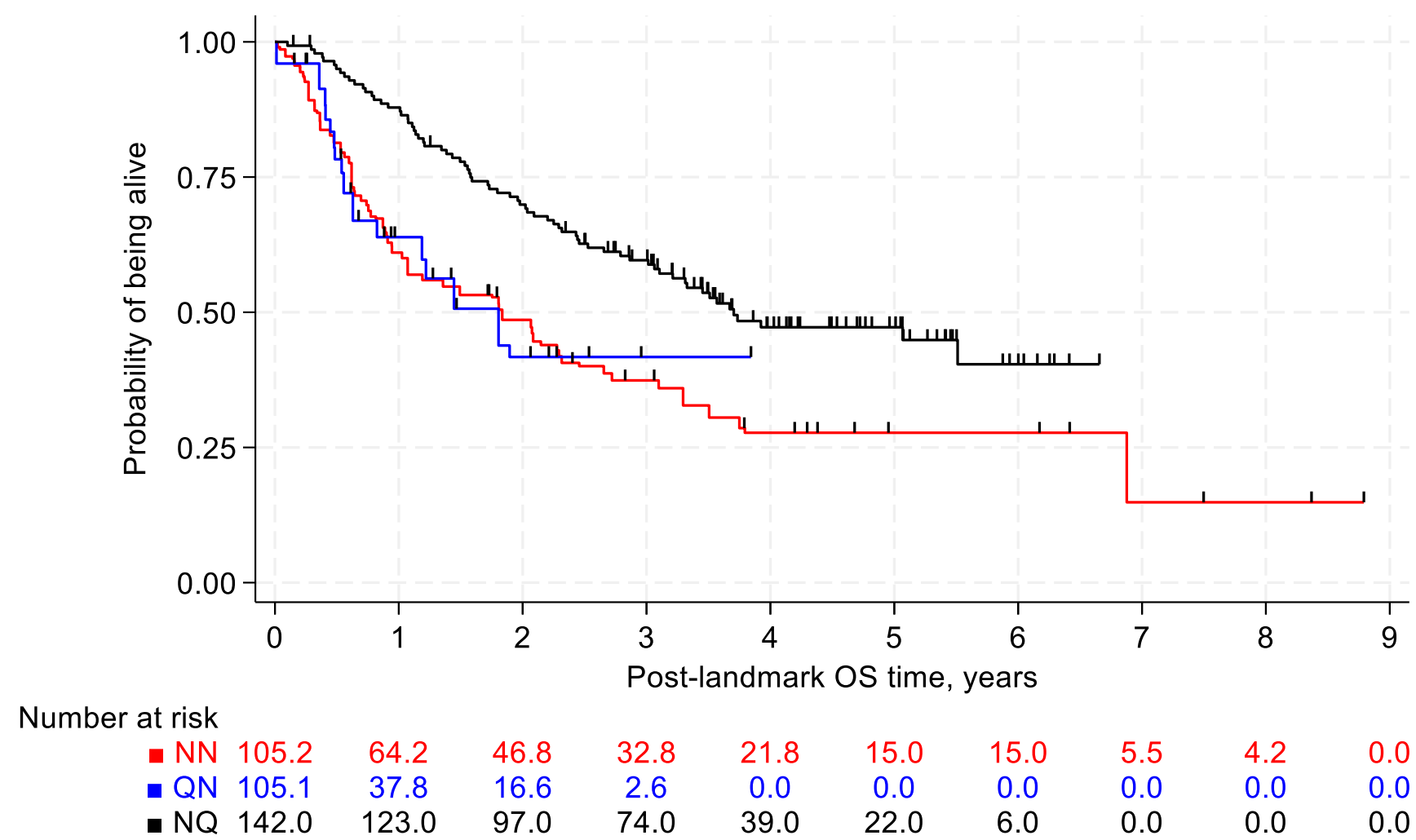	see below

Cox model (2):

	HR (95% CI)	p Value
QN vs NN	1.09 (0.66 to 1.82)	0.730
NQ vs NN	**0.41** (0.29 to 0.58)	**<0.001**

Cox model (3):

	HR (95% CI)	p Value
QN vs NN	1.10 (0.57 to 2.11)	0.784
NQ vs NN	**0.54** (0.36 to 0.82)	**0.004**

This is a provisional file, not the final typeset article

	Kaplan-Meier plot	Cox model

4) NQ cohort: patients using IL-2 were excluded

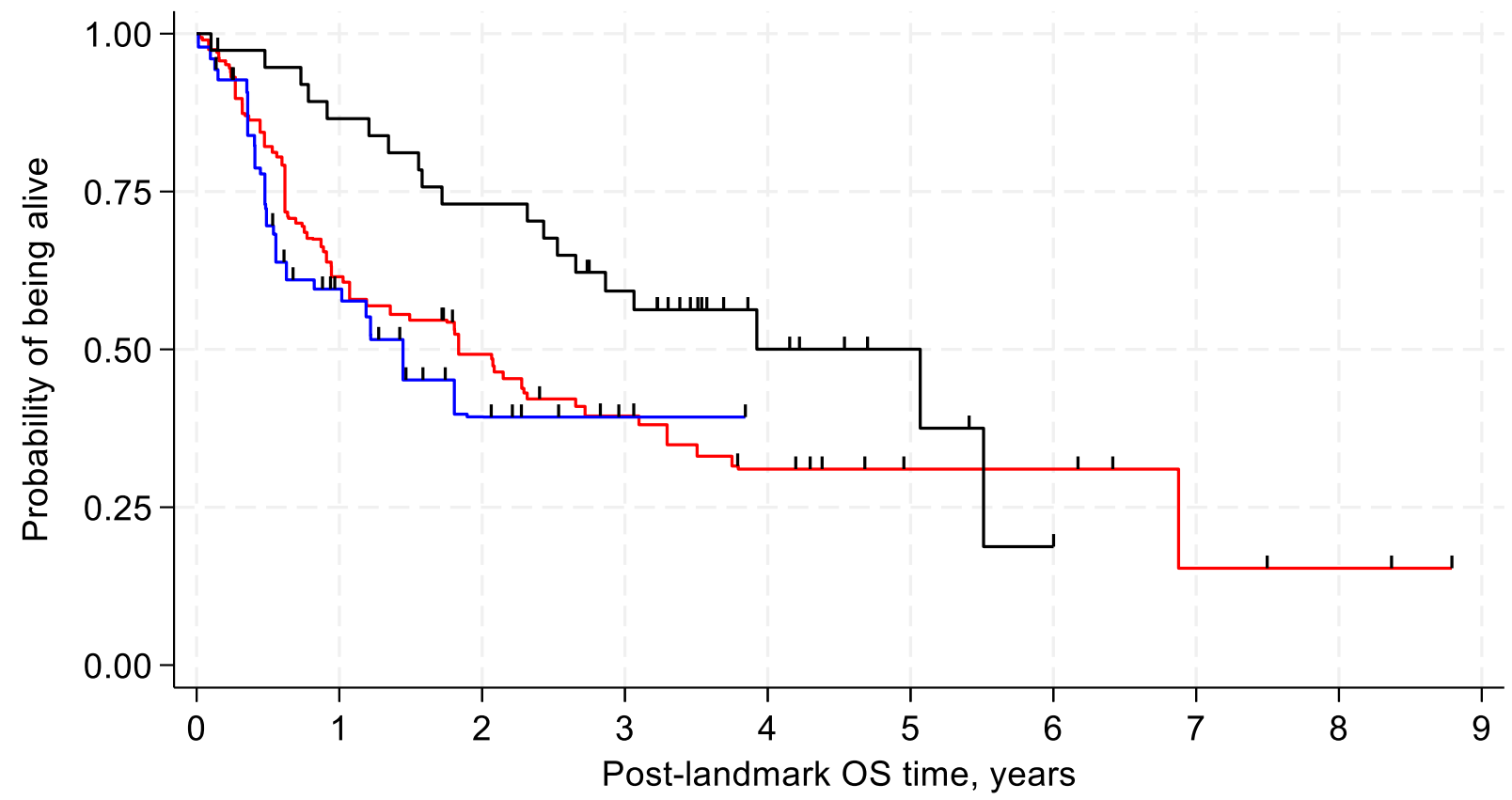

Number at risk

NN	25.3	15.6	11.3	8.6	6.5	4.7	4.7	1.4	1.0	0.0
QN	23.8	7.8	3.3	0.1	0.0	0.0	0.0	0.0	0.0	0.0
NQ	38.0	32.0	27.0	20.0	8.0	4.0	0.0	0.0	0.0	0.0

	HR (95% CI)	p Value
**QN vs NN**	1.54 (0.76 to 3.14)	0.230
**NQ vs NN**	**0.61** (0.33 to 1.11)	0.106
**Other comparisons**		
**NQ vs QN**	**0.39** (0.20 to 0.77)	**0.004**

5) Index date estimated based on the time since the diagnosis

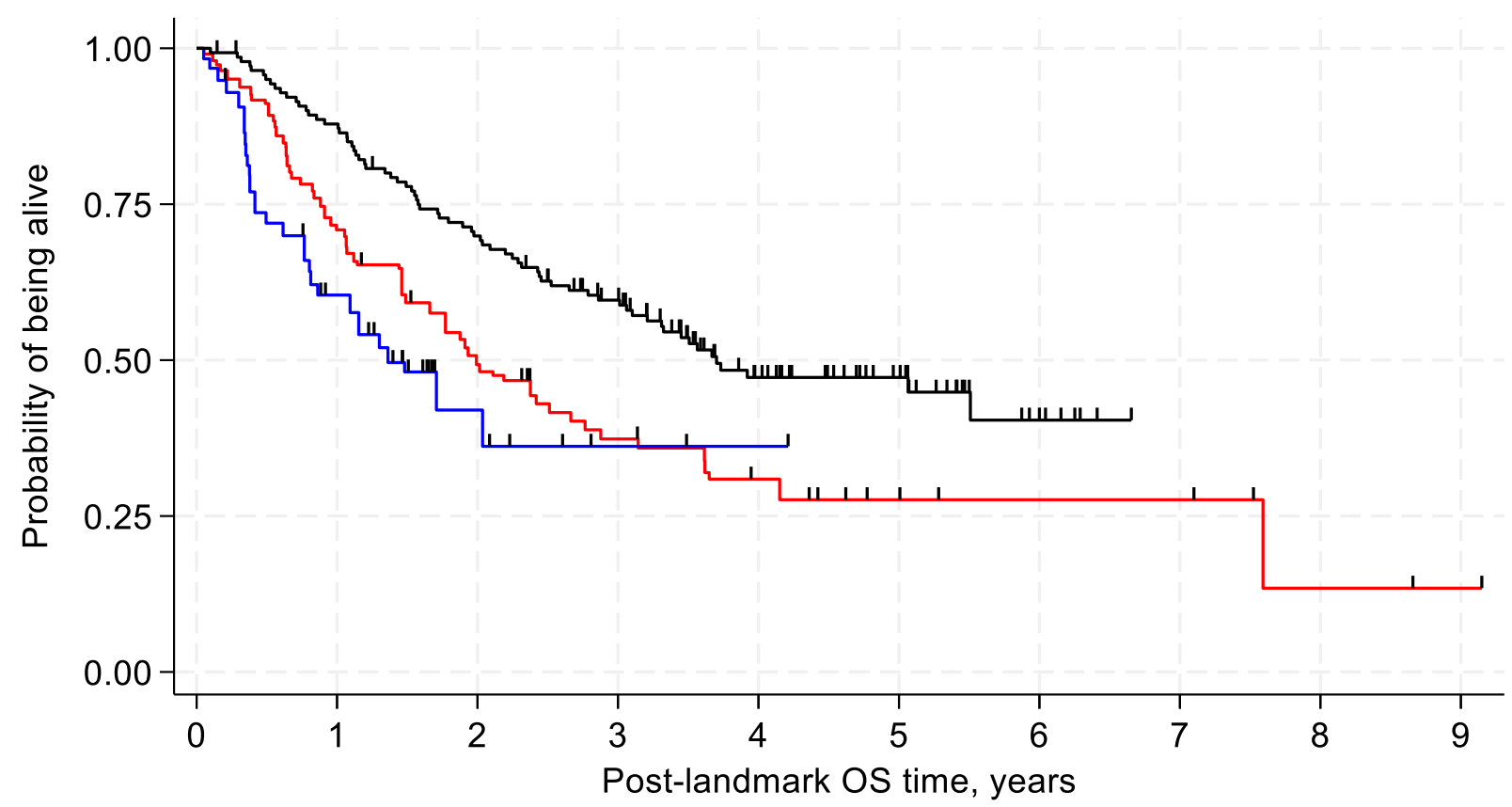

Number at risk

NN	106.5	75.5	49.6	33.4	25.1	17.2	12.0	12.0	4.1	2.0
QN	103.6	52.4	14.2	4.2	1.9	0.0	0.0	0.0	0.0	0.0
NQ	142.0	123.0	97.0	74.0	39.0	22.0	6.0	0.0	0.0	0.0

	HR (95% CI)	p Value
**QN vs NN**	1.42 (0.81 to 2.48)	0.219
**NQ vs NN**	**0.59** (0.40 to 0.88)	**0.010**

	Kaplan-Meier plot	Cox model

6) NN cohort: R1 control only

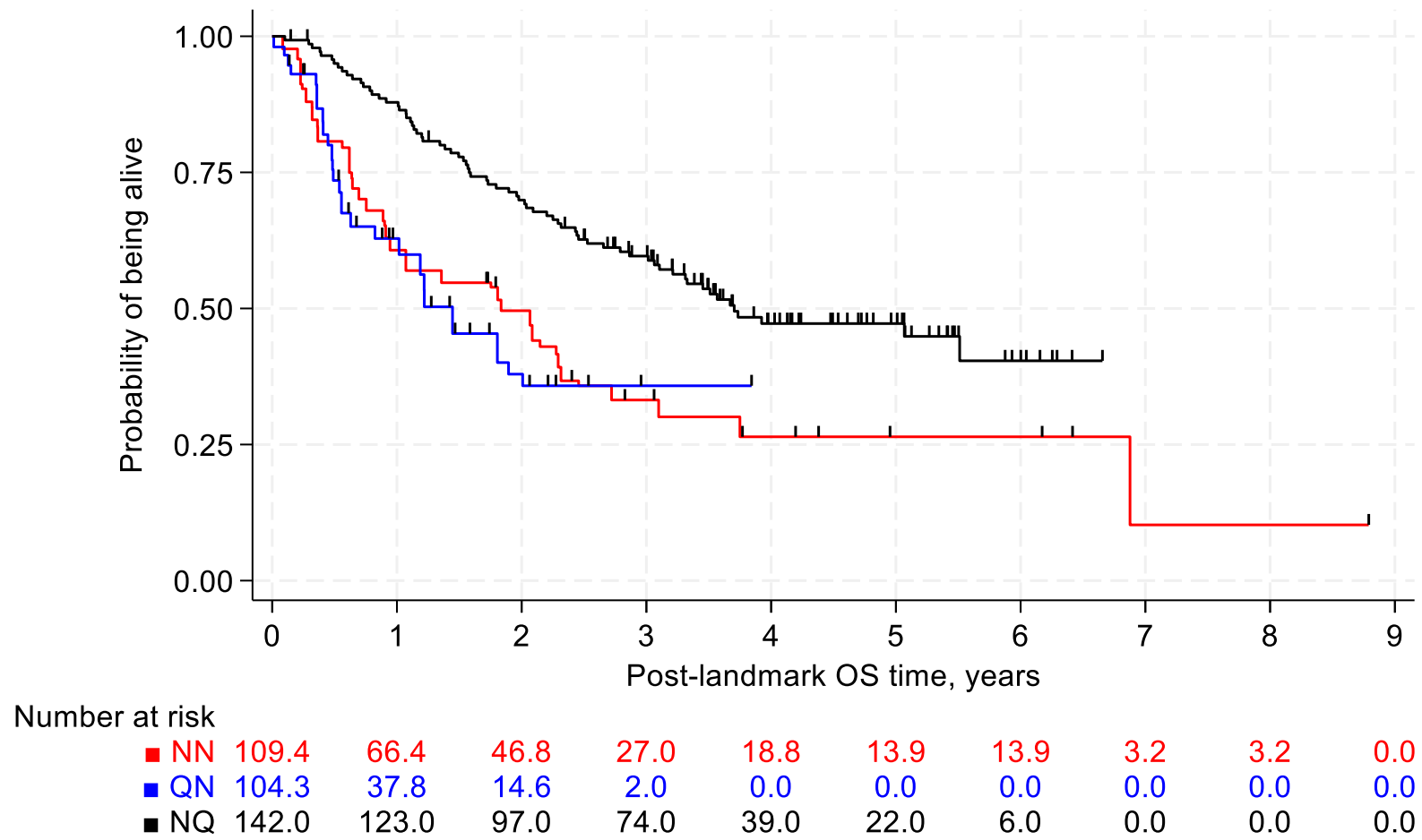

	HR (95% CI)	p Value
QN vs NN	1.25 (0.66 to 2.34)	0.493
NQ vs NN	**0.52** (0.32 to 0.83)	**0.006**

7) Multivariable Cox model fitted to unweighted post-landmark survival data

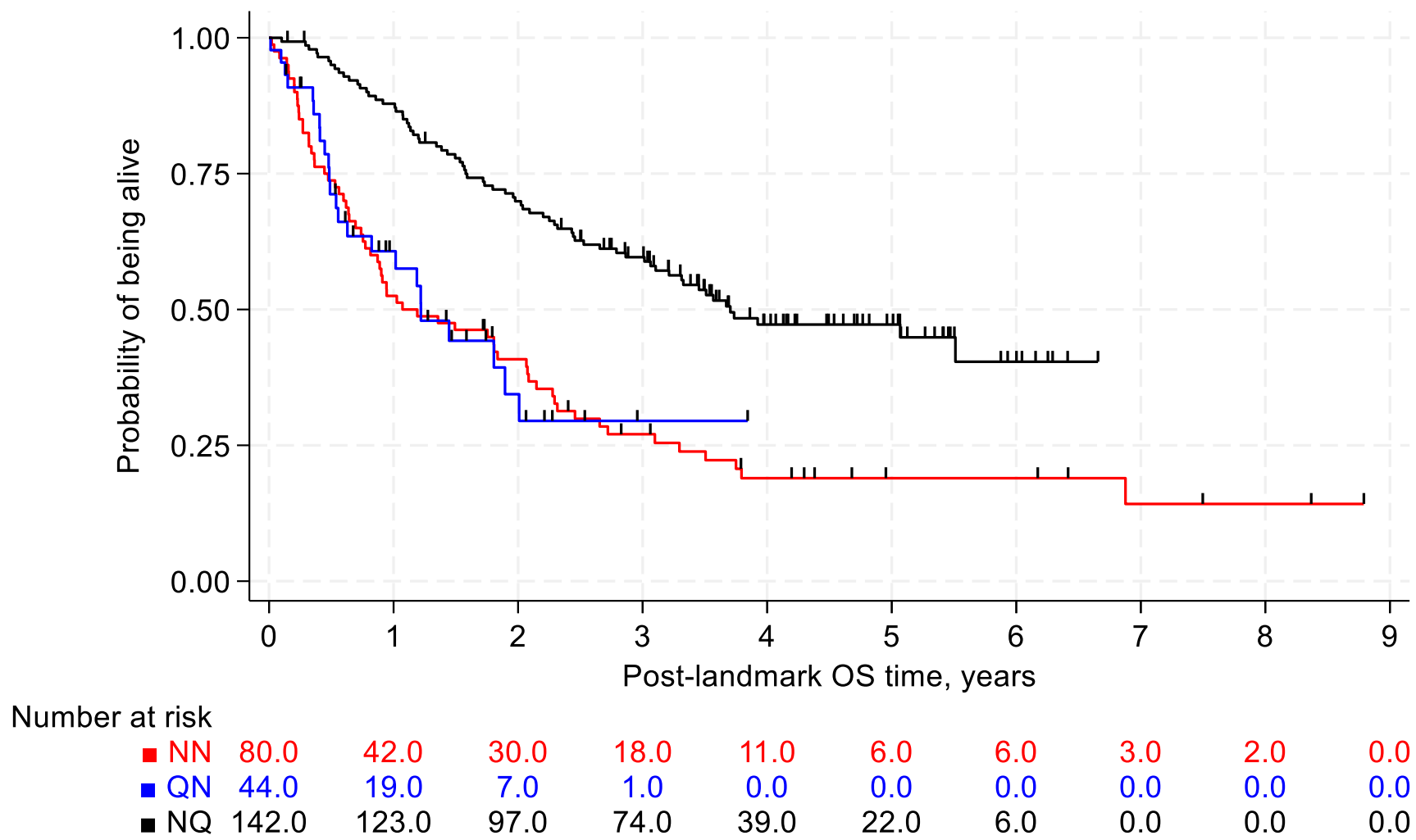

	HR (95% CI)	p Value
QN vs NN	1.18 (0.72 to 1.94)	0.511
NQ vs NN	**0.47** (0.32 to 0.68)	**<0.001**
**Other comparisons**		
NQ vs QN	**0.40** (0.24 to 0.65)	**<0.001**

CI – confidence interval; HR – hazard ratio; NN - no DB in fist-line → no DB after relapse cohort; NQ - no DB → DB cohort; QN - DB → no DB cohort. NN - no DB in fist-line no DB after relapse cohort; NQ - no DB DB cohort; QN - DB no DB cohort

This is a provisional file, not the final typeset article
